# RIP3 Contributes to Cardiac Hypertrophy by Influencing MLKL-Mediated Calcium Influx

**DOI:** 10.1155/2022/5490553

**Published:** 2022-04-14

**Authors:** Honghong Xue, Hongtao Shi, Fan Zhang, Hao Li, Chao Li, Qinghua Han

**Affiliations:** ^1^Department of Cardiology, The First Hospital of Shanxi Medical University, Taiyuan, Shanxi 030001, China; ^2^Shanxi Medical University, Taiyuan, Shanxi 030001, China

## Abstract

Receptor-interacting protein 3(RIP3), a RIP family member, has been reported as a critical regulator of necroptosis and involves in the pathogenesis of various heart diseases. However, its role in the development of myocardial hypertrophy after pressure overload is unclear. We aimed to investigate the roles of RIP3 in pathological cardiac hypertrophy. A rat model of myocardial hypertrophy induced by the aortic banding method was used in this study. Neonatal rat cardiomyocytes (NRCMs) were stimulated with angiotensin II (Ang-II) or phenylephrine (PE) to induce neurohumoral stress. Our results showed that RIP3 level was significantly elevated in the hypertrophic myocardium tissues from patients, rats subjected to AB surgery, and NRCMs treated with Ang-II or PE. After downregulation of RIP3 expression in NRCMs, the phenotypes of myocardial hypertrophy were obviously alleviated. In mechanism, we demonstrated that RIP3 interacts with mixed lineage kinase domain-like protein (MLKL) and promotes its cell membrane localization to increase the influx of calcium within cells, thereby mediating the development of myocardial hypertrophy. More interestingly, we found the blockage of calcium influx by 2-aminoethoxydiphenyl borate, and lanthanum chloride efficiently reverses RIP3-induced cardiac remodeling in NRCMs. Taken together, our findings indicate a key role of the RIP3-MLKL signaling pathway in myocardial hypertrophy, which may be a novel promising treatment strategy for myocardial hypertrophy.

## 1. Introduction

Due to the aging of the general population, the overall incidence of heart failure has increased, causing a huge clinical and economic burden worldwide [1, 2]. Myocardial hypertrophy is associated with a significantly increased risk of heart failure, and it ultimately progresses to heart failure [3]. Myocardial hypertrophy is an adaptive response of the heart when volume load and pressure load increase. Previously, various cues including neurohumoral activation, energy metabolism imbalance, oxidative stress, and other factors were reported to participate in the pathogenesis of myocardial hypertrophy via influencing signaling pathways such as protein kinase signal transduction pathway, *β*-adrenergic receptor signaling, and Ca^2+^/calmodulin-dependent kinase II signaling [4, 5]. However, exactly the mechanisms underlying myocardial hypertrophy have not yet been fully elucidated, which brings difficulties to the treatment strategies specific to myocardial hypertrophy.

Receptor-interacting protein 3 (RIP3), a member of the RIP family, has specific serine/threonine kinase activity and is widely expressed in a large number of mature tissues such as pancreas, gastrointestinal, lung, liver, kidney, and heart [6] and participates in the process of necrotic apoptosis [7]. RIP3 shows high expression in the hearts of murine and humans, which implies its critical role in the physiological function of the myocardium [8, 9]. As a key regulatory protein of programmed necrosis, RIP3 has been proposed as a participant in the pathogenesis of heart diseases such as vascular atherosclerosis, ischemia-reperfusion injury, myocardial infarction, and heart failure [10–12]. Hu et al. found that the plasma RIP3 concentration of patients with heart failure is significantly higher than that of healthy subjects, and the increase in plasma RIP3 is a predictor of poor prognosis in HF [13]. Zhang et al. indicate that RIP3 deficiency blocks *I*/*R* (or *H*/*R*) and Dox-induced myocardial necroptosis by preventing CaMKII activation [14]. However, it has not been studied whether the expression level of RIP3 is related to the occurrence and development of myocardial hypertrophy. Mixed lineage kinase domain-like protein (MLKL) is the substrate of RIP3, and numerous studies have demonstrated that the formation of a complex between RIP3 and MLKL is an essential step for inducing programmed necrosis [15, 16]. RIP3 and MLKL are significantly increased in vinblastine-caused myocardial damage [17]. Besides, inhibition of RIP3 and MLKL activation attenuates heart failure [18]. However, it is unclear whether this signaling pathway is involved in myocardial hypertrophy.

In this study, we aimed to explore whether and how RIP3 is implicated in the pathogenesis of cardiac hypertrophy both in patients and animal models. We reported that RIP3 aggravated cardiomyocyte hypertrophy and necrosis-dependent cardiac injury. Targeting the RIP3-MLKL pathway protects the heart from myocardial hypertrophy and heart failure.

## 2. Materials and Methods

### 2.1. Materials

Angiotensin II (Ang-II, ab120183) was purchased from Abcam (USA). Phenylephrine (PE, 1533002, MERCK), lanthanum chloride (LaCl_3,_ 449830, MERCK), and 2-aminoethoxydiphenyl borate (2-APB, D9754, MERCK) were purchased from Sigma-Aldrich Chemical Co. (USA).

### 2.2. Neonatal Rat Cardiomyocyte Culture

Primary cultures of neonatal rat cardiomyocytes isolated from 2-day-old Sprague-Dawley rats were prepared as previously described [19]. In brief, the ventricles were enzymatically digested in 0.125% trypsin-EDTA four times for 15 minutes each. The obtained cells were then incubated in Dulbecco's modified Eagle's medium (Gibco, USA) supplemented with 10% fetal bovine serum (Gibco, USA) in a 5% CO_2_ humidified atmosphere at 37°C. Cardiomyocyte hypertrophy was induced by Ang-II (1 *μ*M) or PE (50 *μ*M) treatment following the previous reports [20] [21] with a brief modification.

### 2.3. Aortic Banding Animal Models and Gene Delivery

Male SD rats (8 to 9weeks old) were used in this study. All experiments were carried out following the Guidelines for the Care and Use of Laboratory Animals and were approved by the local ethics committee.

Rat cardiac hypertrophy model was established by aortic banding (AB) surgery to produce pressure-overload-induced hypertrophy. The animals were randomly divided into groups, weighed, anesthetized (3% sodium pentobarbital, 40 mg/kg), and performed AB or sham operation as previously described [22]. Rats were sacrificed 2, 4, and 6 weeks after AB surgery, and left ventricular tissue was excised for further detection. The hearts and lungs were weighed to calculate the heart weight/body weight ratio (HW/BW, mg/g), lung weight/body weight ratio (LW/BW, mg/g), and HW/tibia length (HW/TL, mg/mm) in each group.

In this study, we manipulated RIP3 expression using a recombinant adeno-associated virus vector (serotype 2, rAAV2). Empty rAAV vector and rAAV vector expressing RIP3 (oe-RIP3) or RIP3-specific small hairpin RNA (shRIP3) were ordered from GeneChem company (Shanghai, China). For intramyocardial injection of adenoviruses, rAAV2 was injected into the left ventricular free wall at four sites (10 *μ*l for each) prior to AB surgical ligation.

### 2.4. Coimmunoprecipitation (Co-IP)

The immunoprecipitation assay was performed as described previously [14]. After treating NRCMs with PE, the cells were lysed with immunoprecipitation buffer (50 mmol/l Tris-HCl [pH 7.5], 150 mmol/l NaCl, 0.5% NP-40) containing protease inhibitor cocktail (Roche, 4693116001, Basel, Switzerland). Next, 100 *μ*l cell lysate was taken out as the input, and the remaining cell lysate was incubated with protein-G magnetic beads and RIP3 antibody at 4°C overnight. After washing 3 times with immunoprecipitation buffer, the protein was eluted with SDS sample buffer for Western blotting. Detect the binding of RIP3 protein and MLKL protein.

### 2.5. Cardiomyocyte Infection

To overexpress RIP3, a modified pLenti-PuroR vector carrying the RIP3-coding gene was generated. An empty vector was used as a control. Furthermore, we used a rat's short hairpin RNA targeting RIP3 to silence RIP3 gene expression. The sh-scramble was used as a nontargeting control. The steps for virus packaging are as follows. The recombinant plasmids were cotransfected with the helper plasmids into HEK293T cells. 72 hours after infection, the culture medium containing the virus was harvested, precipitated by PEG8000, and filtered with a 0.45 *μ*m filter. To increase the virus titer, virus particles were purified by iodixanol. The obtained virus was directly added into the medium of target cells or stored at -80°C for a long time. The sequences for RIP3 shRNA are as follows: shRIP3#1-F: 5′-CCGGTCCATTGACAGAGCTGCCTCCTC GAGGAGGCAGCTCTGTCAATGGATTTTTG-3′, shRIP3#1-R: 5′-AATTCAAAAAGAGGCAGCTCTGTCAATGGACTCGAGTCCATTGACAGAGCTGCCTC-3′; shRIP3#2-F: 5′-CCGGACATCCTGGTACAGGACAAGCTCGAGCTTGTCCTGTACCAGGATGTTTTTTG-3′, shRIP3#2-R:5′-AATTCAAAAACTTGTCCTGTACCAGGATGTCTCGAGACATCCTGGTACAGGACAAG-3′; shRIP3#3-F: 5′-CCGGCCGCCTGCATCTGGAGGAGCCTCGAGGCTCCTCCAGATGCAGGCGGTTTTTG-3′, shRIP3#3-R: 5′-AATTCAAAAAGCTCCTCCAGATGCAGGCGGCTCGAGCCGCCTGCATCTGGAGGAGC-3′.

### 2.6. Immunofluorescence

The cell surface area of the NRCMs was assessed via immunofluorescence staining of actin. NRCM cells were fixed in 4% paraformaldehyde for 30 minutes, washed 3 times in PBS, and then, stained with actin (ab6276, Abcam, USA) or MLKL (PA5-115578, Thermo Fischer Scientific, USA) at 4°C overnight. Then, the sections were washed 3 times for 5 min in PBS and subsequentially incubated with Alexa 488/555 secondary antibody (A32723/A31570, Thermo Fischer Scientific, USA) for 60 min at room temperature. If necessary, the slides were finally mounted with DAPI-Aqueous Fluoroshield (ab104139, Abcam, USA) and were further imaged using ImageJ software.

### 2.7. Detection of Intracellular Calcium Assay

Take an appropriate amount of Fluo-4 AM mother solution and dilute it to 4 *μ*M working solution with PBS. After the required treatment, the cells were briefly washed with 1× PBS three times. Next, the cells were added with Fluo-4 AM working solution for incubation at 37°C for 30 minutes, and then, the fluorescent probe was loaded into the solution. 1 hour later, the fluorescence of Fluo-4 was detected by microscopy.

### 2.8. CRISPR/Cas9-Mediated Genome-Editing in Cell Line

The targeting sequences for single-strand guide RNAs were determined online. The single guide RNA sequence was cloned into PX459_V2.0 (Addgene). H9c2 cells were seeded on a 6-well plate (200,000 cells per well), and they were used for cell transfection. The cell transfection experiment was performed according to the manufacturer's instructions. After 24 h posttransfection, puromycin (typically at 1 *μ*g/ml) was added to enrich positively transfected cells. The puromycin selection typically lasts for 2 days, and then, the cell population was seeded onto a 15-cm dish at very low density (100–500 cells per 15 cm plate), and colonies were allowed to form in the course of 10 d. 15–20 clones were picked for knockout verification.

### 2.9. Cardiac Morphology Analysis

To evaluate the degree of myocardial tissue hypertrophy and fibrosis in different treatment groups, we performed morphological analysis on heart slices. After the rats were sacrificed, the isolated heart tissues were immediately immersed in a 10% potassium chloride solution to ensure that they were stopped in diastole and washed with saline solution. Subsequently, these hearts were fixed in 10% formalin for 12–24 hours and embedded in paraffin. Further, the heart tissues were sections into 5 *μ*m slides. Finally, the sections were subjected to hematoxylin-eosin (HE) staining to evaluate myocyte cellular hypertrophy.

### 2.10. Western Blotting

According to previous studies, total protein was extracted from rat myocardial tissue and cultured cardiac cells with RIPA lysis buffer supplemented by a protease inhibitor cocktail (Complete, Roche). After quantification using the BCA kit (Beyotime Institute of Biotechnology, China), the equal amount of protein samples were separated by SDS-PAGE and then transferred to the PVDF membrane. The membranes were blocked with nonfat milk in 1X TBST for 1 hour at room temperature and then incubated with the primary antibodies at 4°C overnight. The next day, the membranes were washed using 1 X TBST for 3 × 5 min, and then, the membranes were incubated with secondary antibodies (at an optimized concentration) for 1-2 hours at room temperature. Finally, an image analyzer (Bio-Rad) was used to detect and evaluate the band density on the film. The primary antibodies include against RIP3 (PRS2283, Sigma), *β*-MHC (MA1-83347, ThermoFisher Scientific), ANP (PA5-29559, ThermoFisher Scientific), MLKL, ZO-1 (40-2300, ThermoFisher Scientific), GAPDH (PA1-987, ThermoFisher Scientific), and ACTIN (ab6276, Abcam). The secondary antibody used including goat anti-rabbit IgG and goat anti-mouse IgG was purchased from Abcam (ab6789, ab205718, Abcam).

### 2.11. Real-Time Quantification PCR (qPCR)

To examine the mRNA expression of cardiac hypertrophy and fibrosis-related markers, total mRNA was collected using Trizol reagent (ThermoFisher Scientific, USA) according to the manufacturer's instructions. After the synthesis of the strand of cDNA using the Takara kit (RR036A, TaKaRa, China), qPCR was performed using the TB Green Premix Ex Taq (RR420A, TaKaRa, China). Each analysis was performed in three replicates, and the expression levels of target genes were normalized to GAPDH gene expression.

### 2.12. Statistics Analysis

Continuous variables were summarized as mean ± standard error. And all measurement data passed the normal distribution test (*P* > 0.05 by one-sample *K*-*S* test). The comparison of the two groups of means was analyzed by the Student's *t*-test. The difference between the other two groups was through independent sample one-way analysis of variance (ANOVA) followed by a post hoc Tukey test. *P* < 0.05 was used as a criterion of statistical significance.

## 3. Results

### 3.1. The Protein Level of RIP3 Is Elevated in Hypertrophic Hearts

**Figure 1:**
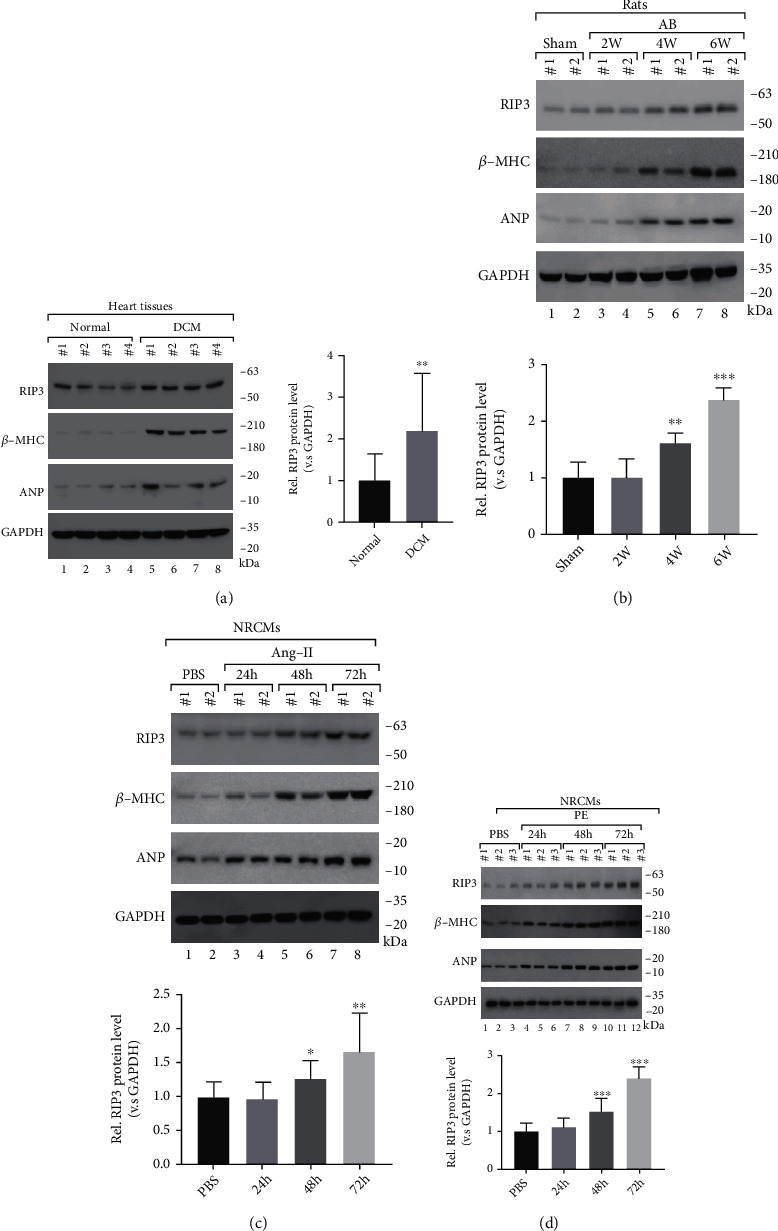
The protein level of RIP3 is elevated in hypertrophic hearts. (a) Representative Western blots and quantitation of RIP3 expression in heart tissue from patients with dilated cardiomyopathy (DCM) and normal controls. (b) (Up) Western blots of RIP3, ANP, *β*-MHC, and GAPDH and (down) relative quantitation of RIP3 expression in heart tissue from rats after sham or AB operation. (c) (Up) Western blots of RIP3, ANP, *β*-MHC, and GAPDH and (down) relative quantitation of RIP3 in NRCMs stimulated with Ang-II (1 *μ*M) or PBS. (d) (up) Western blots of RIP3, ANP, *β*-MHC, and GAPDH and (down) relative quantitation of RIP3 in NRCMs stimulated with PE (50 *μ*M) or PBS. kDa: kilo-Dalton; ^∗^*P* < 0.05, ^∗∗^*P* < 0.01, and ^∗∗∗^*P* < 0.001.

To explore the roles of RIP3 in cardiac hypertrophy, we first determined its protein level in the heart tissues of dilated cardiomyopathy (DCM) patients and normal controls. Intriguingly, we observed that RIP3 in the heart tissues of DCM patients was significantly elevated compared with that in normal controls (Figure 1(a)). Meanwhile, expressions of ANP and *β*-MHC proteins also were highly expressed in DCM heart tissues, confirming the cardiomyopathy phenotype (Figure 1(a)). For the sake of further investigating RIP3's roles in cardiomyopathy, we generated several cardiomyocyte hypertrophy models on rats by aortic banding (AB) surgery and on neonatal rat cardiomyocytes (NRCMs) by angiotensin II (Ang-II) or phenylephrine (PE) treatment. In the rat AB model, since the 4th week after surgery, the expression of RIP3 in myocardial tissue dramatically increased and tightly associated with the expression level of *β*-MHC and ANP (Figure 1(b)), showing its alteration goes along with the pathogenesis of cardiomyopathy. Similarly, both in Ang-II and PE-treated NRCMs, the expression of RIP3 protein was distinguishably increased since the 48th hour after treatment (Figures 1(c) and 1(d)), indicating a remarkable correlation with *β*-MHC and ANP protein levels. Unexpectedly, we found that the mRNA level of RIP3 failed to exhibit significant alteration in the heart tissues of DCM patients, AB rats, and Ang-II or PE-treated cardiomyocytes (Figure S1A–D). Taken together, our data showed that the RIP3 protein level was elevated among multiple cardiac hypertrophy models, and the aberration of RIP3 is caused at the posttranscriptional level.

### 3.2. RIP3 Contributes to Cardiac Hypertrophy In Vitro

**Figure 2:**
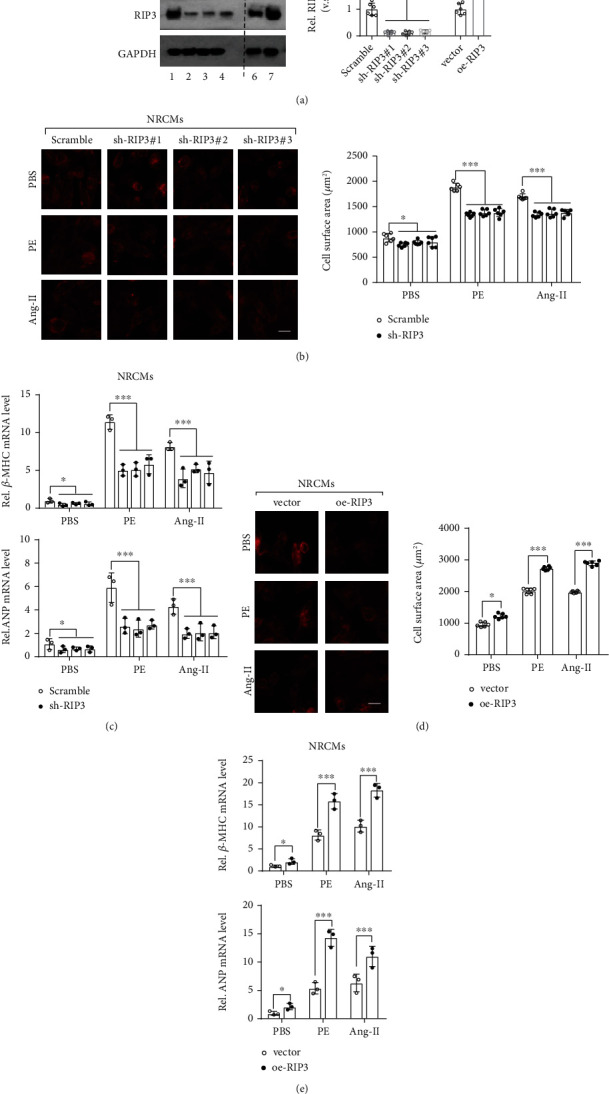
RIP3 contributes to cardiac hypertrophy in vitro. (a) Representative Western blot analysis and relative quantitation of RIP3 in NRCMs stably transfected with scramble (negative control), sh-RIP3, vector (empty), and oe-RIP3 plasmid. (b) Cell morphology of NRCMs was determined by actin staining. NRCMs in (a) (lanes 1-4) were stimulated by Ang-II (1 *μ*M), PE (50 *μ*M), and PBS for 24 hrs. (c) ANP and *β*-MHC mRNA levels were determined by real-time qPCR. NRCMs treated as in (b) were used. (d) Cell morphology of NRCMs was determined by actin staining. NRCMs in (a) (lanes 6-7) were used and stimulated by Ang-II (1 *μ*M), PE (50 *μ*M), and PBS for 24 hrs. (e) ANP and *β*-MHC mRNA levels were determined by real-time qPCR. NRCMs treated as in (d) were used. ^∗^*P* < 0.05, ^∗∗^*P* < 0.01, and ^∗∗∗^*P* < 0.001.

To study whether RIP3 is involved in myocardial hypertrophy, we modified RIP3 expression using a RIP3-targeting shRNA(shRIP3) to downregulate RIP3 in NRCMs and then evaluated the cell's response to Ang-II and PE-induced cardiomyocyte hypertrophy. We found that shRIP3 significantly reduced the protein level of endogenous RIP3 (Figure 2(a), lanes 1-4). In RIP3 knockdown cardiomyocytes, the cell surface area of cardiomyocytes was significantly smaller than that of the control group (Figure 2(b)). Consistently, the mRNA expression of ANP and *β*-MHC was substantially decreased in RIP3 knockdown cardiomyocytes after Ang-II and PE treatment (Figure 2(c)). In another line, we also overexpressed RIP3 in NRCMs (Figure 2(a), lanes 6-7). Interestingly, our observations showed that ectopic RIP3 obviously enhanced Ang-II and PE-induced cardiac hypertrophy in vitro (Figure 2(d)). Also, we noticed that, in RIP3-overexpressed NRCMs, the upregulation of ANP and *β*-MHC mRNA stimulated by Ang-II and PE was further enhanced (Figure 2(e)). These data altogether demonstrate that RIP3 contributes to cardiac hypertrophy in vitro. RIP3 decreases alleviated cardiac hypertrophy.

### 3.3. RIP3 Contributes to Cardiac Hypertrophy In Vivo

**Figure 3:**
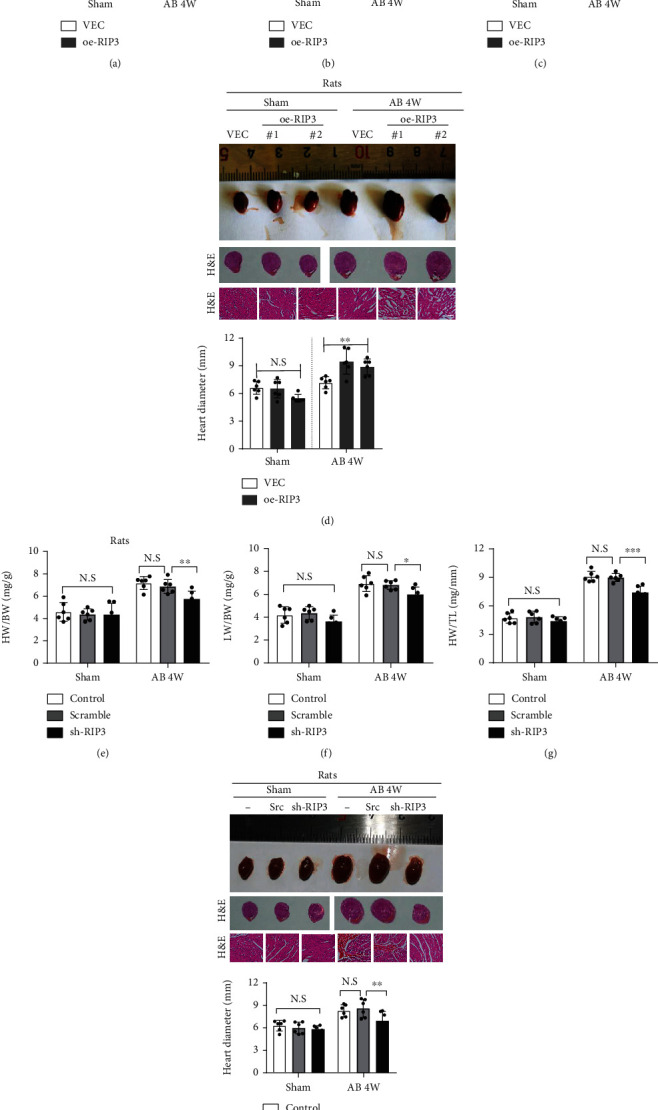
RIP3 contributes to cardiac hypertrophy in vivo. (a–c) Heart weight/body weight ratio (HW/BW), lung weight/body weight ratio (LW/BW), and HW/tibia length (HW/TL, mg/mm) of the indicated rats. Rats were infected with RIP3-containing or VEC (empty vector) rAVVs and then underwent sham or AB surgery. 4 weeks later, these parameters of rats were determined. (d) The gross appearance, size, and hematoxylin-eosin (H&E) staining of the whole heart from cardiomyocyte-specific oe-RIP3 and VEC rats. The rats as in (a) were used, which received sham or AB surgery, respectively. (e–g) Heart weight/body weight ratio (HW/BW), lung weight/body weight ratio (LW/BW), and HW/tibia length (HW/TL, mg/mm) of the indicated rats. The control rats and rats infected with scramble or shRIP3-containing rAVVs were subjected to sham or AB surgery, and 4 weeks later, the indicated parameters were determined. (h) The gross appearance, size, and H&E staining of the whole heart from rats in (e–g). -: control; Scr: scramble. *n* = 6 per experimental group, ^∗^*P* < 0.05, ^∗∗^*P* < 0.01, and ^∗∗∗^*P* < 0.001.

To study the functional role of RIP3 in cardiomyocytes during cardiac hypertrophy, we overexpressed RIP3 in rats using adeno-associated virus vector, and then, RIP3-overexpressed rats and control rats were subjected to AB surgery. 4 weeks after AB surgery, the heart weight/body weight (HW/BW) ratio, lung weight/body weight (LW/BW) ratio, and HW/tibia length (HW/TL) ratio were calculated to reflect cardiac hypertrophy. Compared with the control group, HW/BW, LW/BW, and HW/TL of oe-RIP3 rats were significantly increased (Figures 3(a)–3(c)), but no significant changes were observed in the sham operation group. Meanwhile, histological analysis showed that, after AB operation, the cross-sectional areas of myocardial cells, fibrosis, and heart diameter of oe-RIP3 rats were significantly larger than those of control rats (Figure 3(d)). Subsequently, we downregulated the expression of RIP3 in rat heart tissues by introducing RIP3 specific shRNA rAVVs into rats and then carried out AB surgery on them. Interestingly, we found that, after AB surgery, HW/BW, LW/BW, and HW/TL of the shRIP3 group were significantly reduced when compared with the scramble group, while no significant alterations were observed in the sham operation group (Figures 3(e)–3(g)). Histological analysis also showed that after AB operation in shRIP3 rats, the cross-sectional area of fibrosis and heart diameter of myocardial cells was significantly smaller than that of the shRNA scramble group (Figure 3(h)). These data indicate that overexpressing RIP3 in cardiomyocytes appears to aggravate but not initiate pathological myocardial hypertrophy.

### 3.4. RIP3 Is Implicated in the MLKL-Mediated Calcium Influx

**Figure 4:**
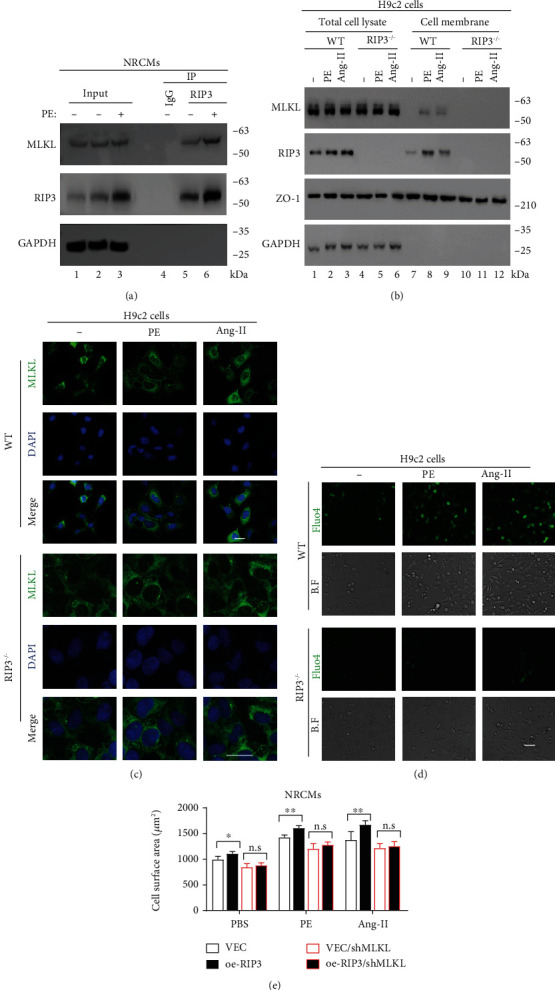
RIP3 is implicated in the MLKL-mediated calcium influx. (a) Co-IP of RIP3 and MLKL in NRCMs stimulated with PE. (b) Western blot showed the cell membrane fraction assay of WT and RIP3^−/−^ H9c2 cells, RIP3^−/−^, RIP3 knockout. WT and RIP3^−/−^ H9c2 cells were stimulated with Ang-II (1 *μ*M), PE (50 *μ*M), and PBS for 24 hrs. (c) Immunofluorescence of MLKL in WT and RIP3^−/−^ H9c2 cells stimulated with Ang-II or PE. The same cells in (b) were used. (d) Intracellular calcium concentration of H9c2 cells was determined by Fluo4 staining. The same cells in (c) were stimulated with Ang-II (1 *μ*M) or PE (50 *μ*M) for 24 hrs. (e) The surface area of the indicated NRCM cell lines. Cells were stimulated with Ang-II (1 *μ*M) or PE (50 *μ*M) for 24 hrs. VEC: infected with vector plenti; VEC/shMLKL: infected with vector/shMLKL plenti; oe-RIP3: infected with RIP3 plenti; oe-RIP3/shMLKL: infected with RIP3/shMLKL plenti. kDa: kilo-Dalton; ^∗^*P* < 0.05, ^∗∗^*P* < 0.01, and ^∗∗∗^*P* < 0.001.

In order to clarify the molecular mechanism of the hypertrophic response of cardiomyocytes induced by RIP3, we evaluated the intracellular signals of isolated NRCMs stimulated by Ang-II and PE. Previous studies have shown that RIP3 physically interacts with MLKL and then MLKL translocates to the cell membrane, which promotes the influx of calcium ions and leads to cell necrosis [23]. Therefore, we conducted Co-IP experiments using NRCMs to explore whether PE treatment influences the interaction between endogenous RIP3 and MLKL. Interestingly, our data revealed that MLKL interacts directly with RIP3, which also was enhanced by PE treatment (Figure 4(a)). To determine whether RIP3 influences the subcellular localization of MLKL protein, we carried out a cell membrane fraction assay using WT and RIP3^−/−^ H9c2 cells. There was no difference in the protein levels of MLKL in WT and RIP3^−/−^ cell lysates after PE or Ang-II stimulation, but MLKL expression was absent on the RIP3^−/−^ H9c2 membrane (Figure 4(b) and S2A). Furthermore, we performed immunofluorescence using MLKL antibody to directly observe its subcellular localization alteration in RIP3^−/−^ H9c2 cells after PE or Ang-II treatment. Compared with the WT H9c2 cells, MLKL proteins in RIP3^−/−^ cells departed from the cell membrane surface (Figure 4(c)). More importantly, we observed that the elevation of calcium concentration by PE or Ang-II treatment was abated in RIP3^−/−^ H9c2 cells, determined by Fluo4 staining (Figure 4(d)). In cell morphology, our results showed that loss-function of MLKL by shRNA largely blocked the cell surface increase induced by RIP3 overexpression in the context of PE or Ang-II treatment (Figure 4(e)). These results suggest that RIP3 interacts with MLKL to form complex and localize on the cell membrane, promoting intracellular calcium influx, and MLKL is necessary for RIP3 to achieve its biological functions.

### 3.5. Blockage of Calcium Influx Reverses RIP3-Mediated Exacerbation of Cardiac Remodeling

**Figure 5:**
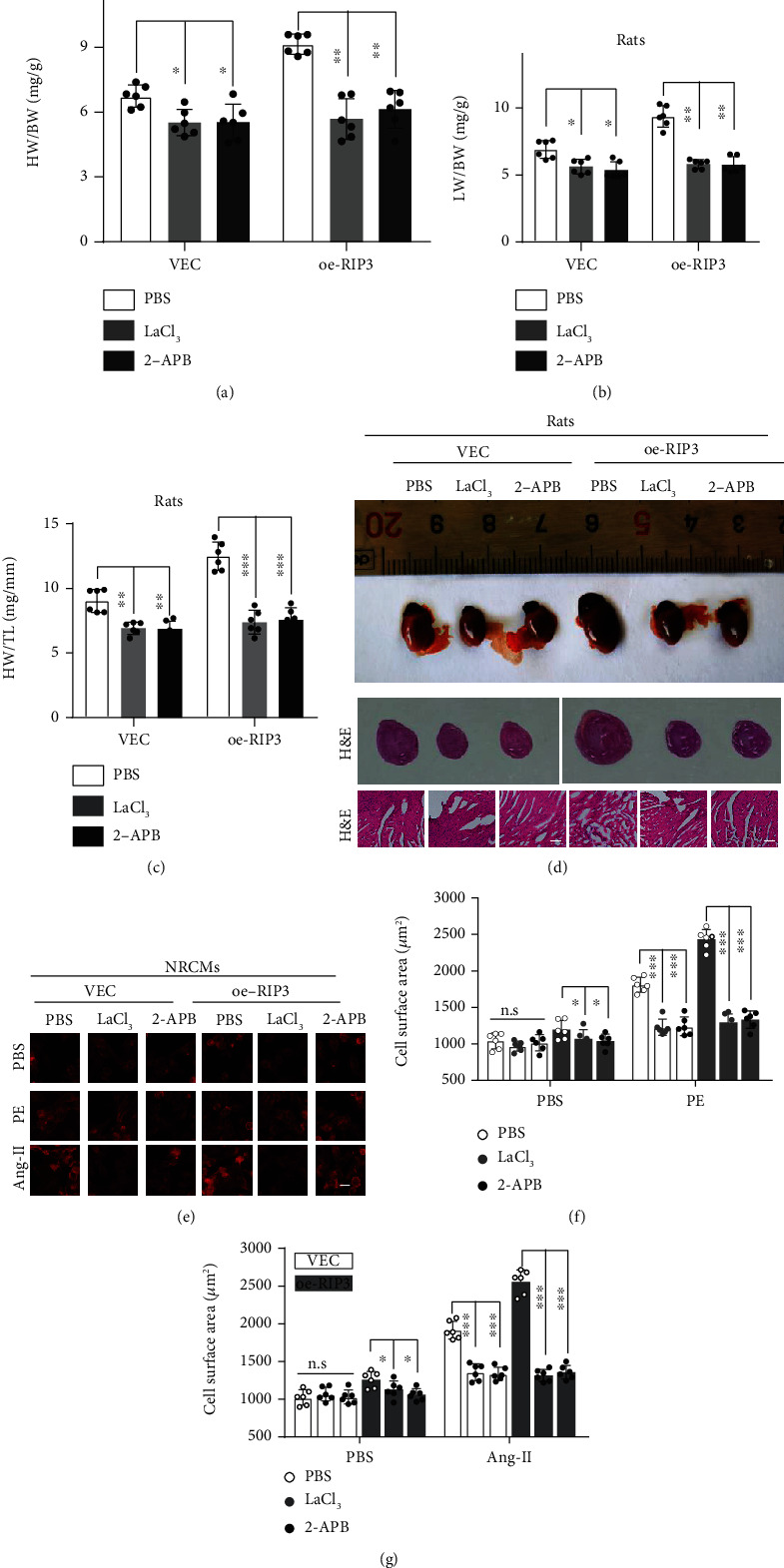
Blockage of calcium influx reverses RIP3-mediated exacerbation of cardiac remodeling. (a–c) Heart hypertrophy parameters including HW/BW, LW/BW, and HW/TL of rats. Rats (VEC and oe-RIP3) were treated with PBS, LaCl_3_ (5 mg/kg body weight, BW), and 2-APB (5 mg/kg BW) by intraperitoneal for 4 weeks. (d) The gross appearance and H&E staining of the whole heart from the rats in (a–c). (e–g) Cell morphology of NRCMs was determined by actin staining. NRCMs were stably infected with empty (VEC) or RIP3 plasmid, which were prestimulated by LaCl_3_ (2.5 *μ*M) and 2-APB (10 *μ*M) for 24 hrs and then treated with Ang-II (1 *μ*M), PE (50 *μ*M), and PBS for 24 hrs. *n* = 6 per experimental group, ^∗^*P* < 0.05, ^∗∗^*P* < 0.01, and ^∗∗∗^*P* < 0.001.

Next, to determine that blocking calcium influx reverses the deterioration of RIP3-mediated cardiac remodeling, we used a calcium channel blocker-lanthanum chloride (LaCl_3_) and the store-operated calcium channel inhibitor 2-APB to inhibit calcium flow. We found that after treatment with LaCl_3_/2-APB, HW/BW, LW/BW, and HW/TL were significantly lower than those in the PBS group (Figures 5(a)–5(c)). After oe-RIP3, the above indexes decreased more significantly than those in the PBS group (Figures 5(a)–5(c)). Meanwhile, histological analysis showed that the hypertrophy of cardiac tissue and myocardial cells was significantly reduced after LaCl_3_/2-APB treatment when compared with the PBS group (Figure 5(d)). In oe-RIP3 rats, LaCl_3_/2-APB also largely abolished the effect of oe-RIP3 on myocardial cells and heart size (Figure 5(d)). In addition, we pretreated WT and oe-RIP3 NRCMs with LaCl_3_/2-APB and then stimulated them by PE, Ang-II, and PBS to observe the phenotype of cardiomyocyte hypertrophy. Immunofluorescence showed that PE or Ang-II stimulated cardiomyocyte hypertrophy, while oe-RIP3 further enhanced the degree of cardiomyocyte hypertrophy (Figures 5(e)–5(g)). Intriguingly, after treatment with LaCl_3_ or 2-APB, cardiomyocyte hypertrophy induced by oe-RIP3 was obviously reversed both in PE and Ang-II stimulated cells (Figures 5(e)–5(g)). Altogether, calcium channel inhibitors can reverse RIP3-mediated exacerbation of cardiac remodeling, and calcium influx largely mediates RIP3's detrimental roles in cardiac remodeling.

## 4. Discussion

The role of RIP3 in inflammation and programmed necrosis has been well established in recent years, but the evidence for its role in the cardiovascular system is limited. Some studies have shown that RIP3-dependent necroptosis leads to cardiac injury and myocardial remodeling [12, 14, 24]. Therefore, we investigated the role and mechanism of RIP3 in myocardial hypertrophy and remodeling. Our results showed that (1) RIP3 protein expression was significantly increased in the pressure- and drug-treated models of myocardial hypertrophy; (2) loss function of RIP3 by shRNAs in rats reduced myocardial hypertrophy, and oe-RIP3 rats increased myocardial hypertrophy; (3) RIP3 interacts with MLKL to localize intracellular membrane-mediated calcium influx, thus promoting cardiomyocyte hypertrophy; and (4) blockage of calcium influx reverses RIP3-mediated exacerbation of cardiac remodeling. Our current research results indicate that RIP3 may be a key factor regulating the occurrence and development of myocardial hypertrophy.

Previous studies have shown that necrosis or apoptosis is the main cause of myocardial cell death. In recent years, necroptosis is considered to be another important mediator of cell death in cardiovascular diseases [25]. Necroptosis is a newly discovered mode of cell death that does not depend on caspase, with typical necrotic performance and gene regulation [26, 27]. Unlike other mechanisms, it involves active cell death triggered by specific signaling pathways, rather than nonspecific damage. Necroptosis is involved in the pathogenesis of many diseases, including neurological diseases [28], malignant tumors [29], and inflammatory diseases [30]. In addition, necroptosis also plays an important role in cardiovascular disease. Factors such as RIP3 and MLKL play an important role in the process of procedural necrosis. RIP3 is the decisive initiating factor leading to necrosis. RIP3 plays a vital role in heart damage caused by ischemia and oxidative stress. Basic research reports that RIP3-mediated necrosis occurs in a wild-type mouse ischemia-reperfusion model [31]; interference with RIP3 can alleviate myocardial cell necrosis in mice [32]. The necroptotic pathway has been shown to be related to vascular disease. Compared with normal arteries, the expression of RIP3 and MLKL mRNA was significantly increased in advanced atherosclerotic plaques, and RIP3^−/−^ mice are protected from the development of atherosclerosis [10]. Clinical studies have found that the heart-specific overexpression of RIP3 can make the structural activation of necrotic cells aggravate the death of necrotic cardiomyocytes, postMI cardiac remodeling, and cardiac dysfunction [33]. And the expression level of RIP3 is significantly increased in patients with heart failure, and the increase in RIP3 transcription is related to the poor prognosis of heart failure [13, 34]. Our data shows that RIP3 plays a role in the regulation of cardiomyocytes in the process of myocardial hypertrophy. This result enriches the influence of RIP3 on heart disease. However, in the manipulation of RIP3 expression in vivo, only rAAV2-based vectors (for both shRIP3 and oe-RIP3) were utilized, which may be a limitation of the current study due to the possible off-target of this kind of virus.

MLKL is the downstream target of RIP3. After the necrosome is formed, RIP3 can recruit and activate MLKL through phosphorylation of threonine 357 and serine 358 sites of MLKL [16]. Phosphorylated MLKL is converted from monomer to oligomer. The oligomerized MLKL is translocated to cell membranes and organelle membranes and combines with phospholipids, causing the integrity of the membrane to be destroyed, forming a perforated channel [35, 36]. Some studies believe that after MLKL translocates to the cell membrane, it may be due to the promotion of the influx of calcium, which leads to cell necrosis; others believe that MLKL can promote the influx of sodium, leading to changes in intracellular osmotic pressure and cell swelling, leading to cell necroptosis.

Abnormal Ca^2+^ is the core mechanism of various pathophysiological changes such as arrhythmia, myocardial hypertrophy, and heart failure [37]. Intracellular Ca^2+^ homeostasis is the basis for regulating cardiac contractile function. As the second messenger in the cell, Ca^2+^ plays an important role in the signal transduction pathway in the cell. The occurrence and development of cardiomyocyte hypertrophy are often accompanied by abnormal intracellular Ca^2+^ concentration, which in turn causes a series of physiological and pathological reactions [38]. Ca^2+^ enters the cell via type-L calcium channels, which increases Ca^2+^ concentration near calcium-binding sites outside the sarcoplasmic reticulum and triggers the opening of Ca^2+^ release channels of ryanodine receptors on the sarcoplasmic reticulum through a mechanism called Ca^2+^-induced Ca^2+^ release [39]. In the early stage of myocardial hypertrophy, Ca^2+^ influx increases, and the mobilization of Ca^2+^ in myocardial cells is enhanced to maintain the basic functions of the heart [40]. The continuous increase in the influx of Ca^2+^ further enhances the loading and release of SR Ca^2+^, leading to increased intracellular Ca^2+^ levels and increased binding of Ca^2+^ with inactive calmodulin to form active calmodulin [41]. Activated calmodulin is one of the key regulators in the signaling pathway that promotes cardiac hypertrophy. Of course, during this experiment, we only paid attention to the changes in calcium ions caused by MLKL localization on the cell membrane and did not detect whether the myocardial hypertrophy caused by MLKL was caused by Na^+^ changes. This is one of the shortcomings of our experiment.

Currently, the disease-modifying drug for cardiac hypertrophy is still lacking in clinics due to the underlying complex pathogenic mechanisms. Our research shows that the RIP3-MLKL-Ca^2+^ axis could be implicated in the pathogenesis of cardiac hypertrophy. Recently, Qiao et al. reported that inhibition of RIP3 by GSK872 significantly alleviates high glucose fat-induced myocardial fibrosis [42]. Together with our results, we consider that RIP3 kinase inhibitors could be a potential clinical intervention for cardiac hypertrophy. Meanwhile, we observed that LaCl_3_ and 2-APB treatment in a low dosage ameliorate heart hypertrophy phenotype induced by oe-RIP3 on rats. Atrial fibrillation is a progressive disease that has been revealed a variety of myocyte remodeling processes by clinical and animal experimental evidence. Interestingly, Brundel et al. found that LaCl_3_ treatment prevents structural changes in a pacing-induced cellular remodeling in a HL-1 myocyte model for atrial fibrillation [43]. In 2021, Shen et al. reported that 2-APB treatment decreases the *I*/*R*-induced adult rat cardiomyocytes death and myocardial infarct size [44]. These findings suggest that LaCl_3_ and (or) 2-APB may possess beneficial effects on cardiovascular diseases. Therefore, our observations provide guidance for the development of new technologies for drug research and diagnosis and treatment of cardiac hypertrophy using LaCl_3_ and 2-APB.

In summary, our findings indicate that the aberration of RIP3 is caused at the posttranscriptional level. Meanwhile, we demonstrated that overexpression of RIP3 is implicated in the pathogenesis of myocardial hypertrophy by promoting MLKL cell membrane localization, which further increases intracellular calcium influx. Besides, our results show the heart hypertrophy induced by oe-RIP3 is reversed by LaCl_3_ and 2-APB treatment. Therefore, our findings highlight the significant role of the RIP3-MLKL-Ca^2+^ axis on heart functions, which may be an attractive target for future therapeutic interventions in myocardial hypertrophy.

## Data Availability

The data used to support the findings of this study are available from the corresponding author upon request.

## Abbreviation

RIP3: Receptor-interacting protein 3
MLKL: Mixed lineage kinase domain-like protein
DCM: Dilated cardiomyopathy
AB: Aortic banding
Ang-II: Angiotensin II
PE: Phenylephrine
NRCMs: Neonatal rat cardiomyocytes
LaCl_3_: Lanthanum chloride
2-APB: 2-Aminoethoxydiphenyl borate
HW: Heart weight
BW: Body weight
LW: Lung weight
TL: Tibia length
HE: Haematoxylin-eosin.

## Supplementary Materials

Supplementary Materials Supplementary Figure 1: the expression level of RIP3 is elevated in hypertrophic hearts. (A) Relative mRNA expression of RIP3 in heart tissue from dilated cardiomyopathy (DCM) and normal patients. (B) Relative mRNA expression of RIP3 in heart tissue from wild-type (WT) rats after AB or sham surgeries. (C, D) Relative RIP3 mRNA expression of NRCMs. Cells were stimulated by (C) Ang-II (1 *μ*M) and (D) PE (50 *μ*M) for 24 hrs. Supplementary Materials Supplemental Figure 2: RIP3 is implicated in the MLKL-mediated calcium influx. (A) Representative Western blots of MLKL expression in heart tissue from dilated cardiomyopathy(DCM) and normal patients.
