# Supplementary material for: RIP3 Contributes to Cardiac Hypertrophy by Influencing MLKL-Mediated Calcium Influx
Source: Oxid Med Cell Longev. 2022 Apr 14;2022:5490553. doi: 10.1155/2022/5490553 (PMC9023175; doi:10.1155/2022/5490553)

Fig S1. expression level of RIP3 is elevated in hypertrophic hearts.

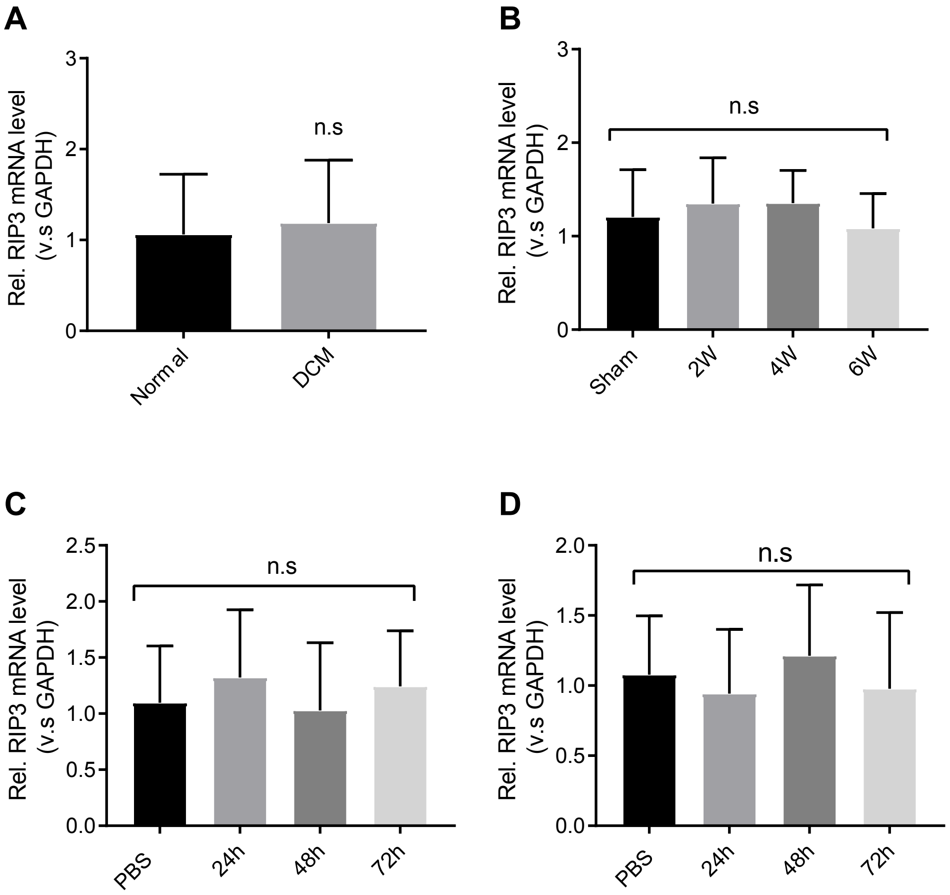

Fig S2. RIP3 is implicated in the MLKL-mediated calcium influx.

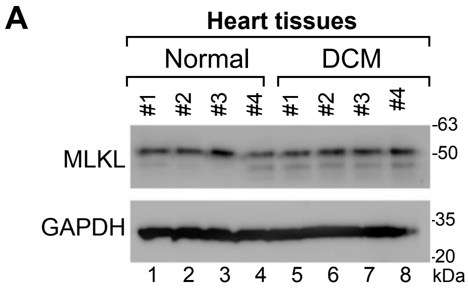

Supplement: Supplementary Materials — Supplementary Figure 1: the expression level of RIP3 is elevated in hypertrophic hearts. (A) Relative mRNA expression of RIP3 in heart tissue from dilated cardiomyopathy (DCM) and normal patients. (B) Relative mRNA expression of RIP3 in heart tissue from wild-type (WT) rats after AB or sham surgeries. (C, D) Relative RIP3 mRNA expression of NRCMs. Cells were stimulated by (C) Ang-II (1 μM) and (D) PE (50 μM) for 24 hrs. Supplementary Materials Supplemental Figure 2: RIP3 is implicated in the MLKL-mediated calcium influx. (A) Representative Western blots of MLKL expression in heart tissue from dilated cardiomyopathy(DCM) and normal patients. [file 5490553.f1.pdf]
